# ViralFlow: A Versatile Automated Workflow for SARS-CoV-2 Genome Assembly, Lineage Assignment, Mutations and Intrahost Variant Detection

**DOI:** 10.3390/v14020217

**Published:** 2022-01-23

**Authors:** Filipe Zimmer Dezordi, Antonio Marinho da Silva Neto, Túlio de Lima Campos, Pedro Miguel Carneiro Jeronimo, Cleber Furtado Aksenen, Suzana Porto Almeida, Gabriel Luz Wallau, on behalf of the Fiocruz COVID-19 Genomic Surveillance Network

**Affiliations:** 1 Department of Entomology and Bioinformatics Core, Aggeu Magalhães Institute-Oswaldo Cruz Foundation (Fiocruz), Campus UFPE-Av. Prof. Moraes Rego s/n, Recife 50670-420, Brazil; 2 Bioinformatics Core, Aggeu Magalhães Institute-Oswaldo Cruz Foundation (Fiocruz), Campus UFPE-Av. Prof. Moraes Rego s/n, Recife 50670-420, Brazil; antonio.marinho@fiocruz.br (A.M.d.S.N.); tulio.campos@fiocruz.br (T.d.L.C.); 3 Oswaldo Cruz Foundation (Fiocruz), Branch Ceará, Eusebio 61760-000, Brazil; pedrom.pm47@alu.ufc.br (P.M.C.J.); cleber.aksenen@gmail.com (C.F.A.); suzanaporto02@gmail.com (S.P.A.)

**Keywords:** genomics, SARS-CoV-2, viruses, virus bioinformatics, genotyping, genomic variants, software

## Abstract

The COVID-19 pandemic is driven by Severe Acute Respiratory Syndrome coronavirus 2 (SARS-CoV-2) that emerged in 2019 and quickly spread worldwide. Genomic surveillance has become the gold standard methodology used to monitor and study this fast-spreading virus and its constantly emerging lineages. The current deluge of SARS-CoV-2 genomic data generated worldwide has put additional pressure on the urgent need for streamlined bioinformatics workflows. Here, we describe a workflow developed by our group to process and analyze large-scale SARS-CoV-2 Illumina amplicon sequencing data. This workflow automates all steps of SARS-CoV-2 reference-based genomic analysis: data processing, genome assembly, PANGO lineage assignment, mutation analysis and the screening of intrahost variants. The pipeline is capable of processing a batch of around 100 samples in less than half an hour on a personal laptop or in less than five minutes on a server with 50 threads. The workflow presented here is available through Docker or Singularity images, allowing for implementation on laptops for small-scale analyses or on high processing capacity servers or clusters. Moreover, the low requirements for memory and CPU cores and the standardized results provided by ViralFlow highlight it as a versatile tool for SARS-CoV-2 genomic analysis.

## 1. Introduction

The emergence [1] and rapid spread of Severe Acute Respiratory Syndrome coronavirus 2 (SARS-CoV-2), the virus that causes the Coronavirus Disease 2019 (COVID-19), and the subsequent establishment of the COVID-19 pandemic [2], triggered a global effort to sequence and identify the circulating SARS-CoV-2 lineages. This effort resulted in the availability of more than five million genomes in the EpiCoV™ database hosted on GISAID in November 2021 [3], representing more than 1600 of the lineages described on PANGO lineages [4].

A range of molecular biology methods have been developed to diagnose SARS-CoV-2 infections, such as RT-qPCRs, RT-LAMP, immunoassays, and Sanger sequencing [5,6,7,8]. However, only whole-genome sequencing can provide enough genetic information (genome-wide mutation patterns) for the reliable lineage discrimination that is necessary for the characterization of variants of concern (VOCs) [9]. Amplicon-based Next-Generation Sequencing (NGS) has become the gold standard methodology for SARS-CoV-2 genome sequencing [10], but the abundance of sequencing data from hundreds or thousands of samples also brings new challenges to bioinformatics analysis. At the moment, the Centers for Disease Control and Prevention (CDC) official git repository contains eighteen bioinformatics tools for dealing with different [11,12,13] SARS-CoV-2 sequencing data [14]. However, even with well-documented workflows that work mostly with nanopore sequencing data [15,16,17], a single workflow that incorporates several key genomic analyses, such as data quality checks, genome assembly, virus lineage assignment, mutation description and intrahost variants variability analysis with short paired-end reads is still lacking.

In this work, we describe a workflow currently used by the Fiocruz COVID-19 Genomic Surveillance Network, which is part of a national effort to characterize and monitor SARS-CoV-2 variants in Brazil [18,19,20]. It was developed to work with paired-end Illumina amplicon sequencing reads and is focused on both pre- and post-genomic analysis. It was designed to support research groups with diverse computational structures, such as personal computers and multi-user servers, through the containerization of the workflow with Docker [21] or Singularity [22].

## 2. Materials and Methods

### 2.1. Worflow Structure

**Figure 1:**
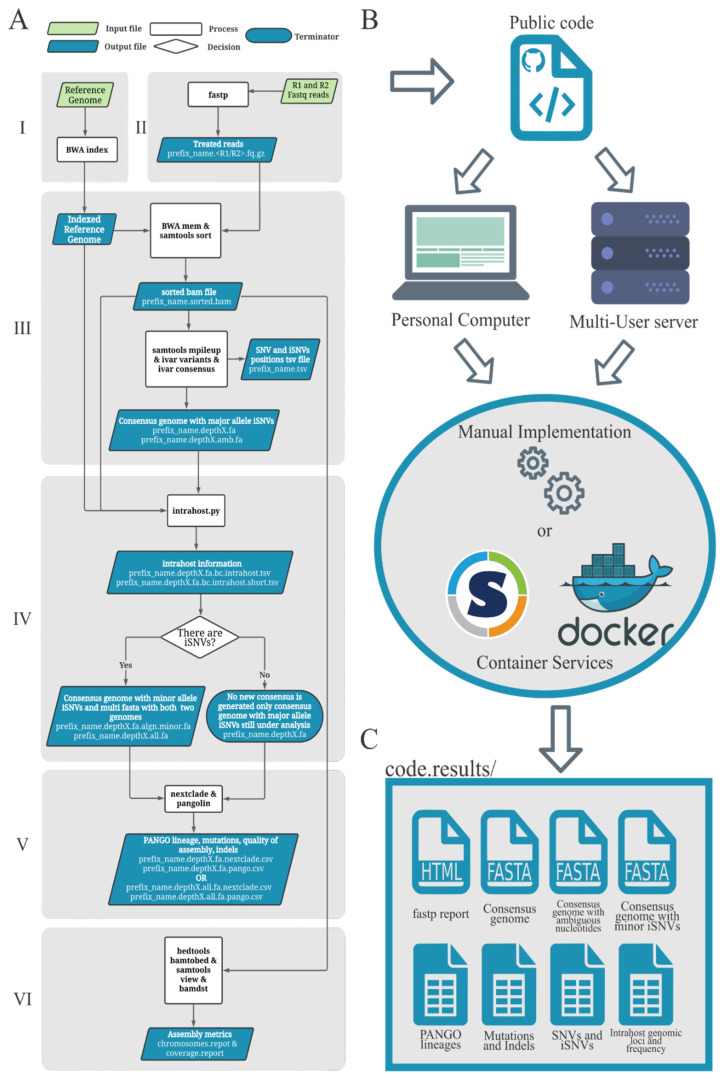
The workflow scheme. (**A**) The six steps of the workflow. (**B**) The workflow can be configured to work on diverse computational environments. (**C**) Some of the most important per sample outputs generated by the workflow.

The workflow was developed within an Ubuntu 20.04.2 LTS Docker environment (https://hub.docker.com/_/ubuntu, accessed on 16 November 2021) and is composed of six steps used to analyze SARS-CoV-2 Illumina paired-end amplicon sequencing data (Figure 1A): reference genome indexing, quality control, consensus generation, intrahost variant analysis, virus lineage assignment and mutation analysis, and assembly metrics analytics. This workflow can be used in different computational environments (Figure 1B).

First, the reference genome is processed and indexes are obtained using the BWA index [23]; we recommend the SARS-CoV-2 reference genome Wuhan-Hu-1, NCBI refseq NC_045512.2 code. In this step, the pangolin tool is updated. The quality control step for the raw sequencing reads is performed with the fastp v.0.20.1 tool [24], where the paired-end reads are trimmed using a minimum read quality threshold (Phred score = 20). The adapters or the PCR primers and the minimum length threshold for the trimmed reads should be defined by the user. In addition to the paired-end treated data, the fastp tool generates an html file information from pre- and post-treatment steps with associated statistics (Figure 1C).

The generation of the consensus genome is performed using a reference-guided assembly strategy. In this step, the paired-end libraries are mapped against a reference genome with BWA v.0.7.17 [23]. Following the alignment step, the Samtools [25] sort and index parameters are used to sort and index the BAM files. Next, minor variant analyses are performed, the Samtools v.1.9 and iVar v.1.3.1 [26] tools are used for the correct recovery of SNPs and indels, and two consensus are generated: one with the majority allele present in every nucleotide position along the genome (iVar consensus -t 0) and another version with ambiguous nucleotide characters, in cases where the majority intrahost Single Nucleotide Variants (iSNVs) encompass up to 60% of allele frequencies (iVar consensus -t 0.6). Only mapped bases with quality equal to or greater than 30 (-q 30) were used in iVar counts. The minimum depth threshold to consider a position with supported intrahost variants can be defined by the user (default equals 100x).

An extra step for intrahost variant calls is necessary, considering that iVar does not provide an option to generate consensus harboring all iSNVs with two or more alleles found in low frequency (≤49% of the reads). Given that the consensus genome with minor iSNVs is essential to understanding the effect of intrahost variants, we developed an *in-house* python script (intrahost_script.py) that uses the allele frequencies per position output of bamreadcount v.0.8.0 [27] to detect only positions with two or more alleles and to generate a consensus harboring all minor supported alleles. To avoid the recognition of sequencing artifacts as intrahost variants, genomic positions were selected:

(i) The minor allele frequency represented at least 5% of the total allele depth;
(ii) The minor alleles had at least 100 reads of depth (default depth);
(iii) The minor allele nucleotides were supported by reads of both senses (at least 5% of depth should come from each read sense).

Combining the first two requirements stated above, a sequenced depth of 2000 reads is required to detect iSNVs present at the minimum frequency of 5%. However, the user can set a different minimum depth threshold, if necessary.

The virus lineages signature is performed with Pangolin. Pangolin and all information about current and new lineages are updated at the moment of the Docker or Singularity image creation to avoid using outdated data and software versions for the analysis. When using interactive containers, the command “pangolin--update” is strongly recommended. The consensus quality and set of mutations are evaluated using nextclade v.0.14.2 [28]. If the analyzed sample shows intrahost signals, Pangolin and nextclade analyses are performed for both consensus versions (with major and minor allele frequencies), while it will run only for a single consensus genome in the absence of iSNV sites. In the last step, the assembly metrics, such as depth and coverage, are extracted with bamdst v.1.0.6 [29].

### 2.2. ViralFlow Scalability 

Infrastructure and computational experience are heterogeneous in the different research groups working with SARS-CoV-2 genomic data; therefore, we evaluated our workflow in two use case scenarios:

- Case I: Using an average personal computer to install all dependencies or using Docker or Singularity container services.
- Case II: Using a multi-user computational server to install all dependencies or using Docker or Singularity container services.

Case I was run on a personal laptop with the following configurations: Ubuntu 20.04.2 LTS, 02 × RAM 8 Gb DDR4 2667 MHz and CPU AMD^®^ Ryzen™ 7-3750H 2.88 GHz. For Case II, we used a computational server with the following configurations: a node with 191 Gb of RAM DRAM 2933 MHz and two CPUs Intel(R) Xeon(R) Gold 5220R CPU @ 2.20 GHz totaling 96 threads. The scalability of ViralFlow according to the number of threads provided was evaluated on both computational resource scenarios using a Singularity container. The performance of the workflow was accessed using two datasets. The first one is a public dataset of 86 Brazilian SARS-CoV-2 Illumina paired-end libraries generated by the amplicon sequencing method using the Illumina COVIDSeq protocol, available under the EMBL-EBI study accession PRJEB47823. It was used to evaluate the computational resources and the run time of the workflow. The second one is an artificial dataset (Supplementary File S1) created with the ART [30] of five paired-end libraries simulating a simultaneous infection (coinfection/codetection) of different SARS-CoV-2 lineages in a single sample (sample information in Table S1), used to evaluate the capacity of the workflow to detect intrahost variants.

### 2.3. Benchmark

To compare the genome assembly coverage breadth, depth and lineage assignment of SARS-CoV-2 lineages, we assembled the same 86 samples with HAVoC [31], a published workflow that performs similar steps of read processing (fastp) and mapping (BWA). We performed two tests with HAVoC:

Case I: fastp with -q (qualified_quality_phred) equal to 20 and parsing the adapters sequence file, to perform the same treatment of ViralFlow

Case II: fastp with -q equal to 15 and without an adapters sequence file, to run the native HAVoC.

Both tests were performed considering a minimum of 5× coverage depth to generate consensus and 75 as the minimum read length threshold in the fastp step.

## 3. Results and Discussion

### 3.1. Performance and Scalability

**Figure 2:**
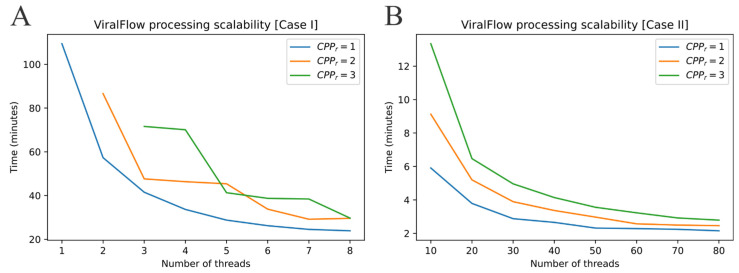
The ViralFlow threads scalability benchmark for (**A**) Case I and (**B**) Case II. CPPr = Cpus per sample requested.

A set of 86 samples was generated in an amplicon-sequencing strategy using the Illumina COVIDSeq Test kit, generating paired-end reads of 150 nucleotides that encompass PCR positive samples for SARS-CoV-2 from the Pernambuco state in Brazil from August 2020 to May 2021 (Table S2). It was assembled and tested in both environments (see the Materials and Methods section, Case I and Case II). The benchmarks for the 86 samples show that it is possible to process this dataset in ~20 min using ≥6 threads (Figure 2A) on a personal computer. Such time can be reduced to ~2 min or less on a dedicated server using ≥50 threads (Figure 2B). Benchmarks also revealed that using more than one thread per sample generally decreases performance. Most of the ViralFlow steps are handled by a single thread, and additional threads imply extra waiting time for those processes to finish. As a general rule of thumb, one thread per sample should scale better in almost every scenario. Therefore, scaling up using a computer/server with a large number of cores/threads is ideal for speeding up ViralFlow results. The total RAM (Random Access Memory) used was ~0.70 Gb and ~0.80 Gb in Case I and Case II, respectively. This number was similar and did not change significantly depending on the number of threads. We observed that the tools used in our workflow relied more on CPU usage than RAM. ViralFlow scales well with the number of threads available and is able to process life-size sequence batches in a few minutes, even using a modest computational infrastructure.

### 3.2. Intrahost Detection

**Figure 3:**
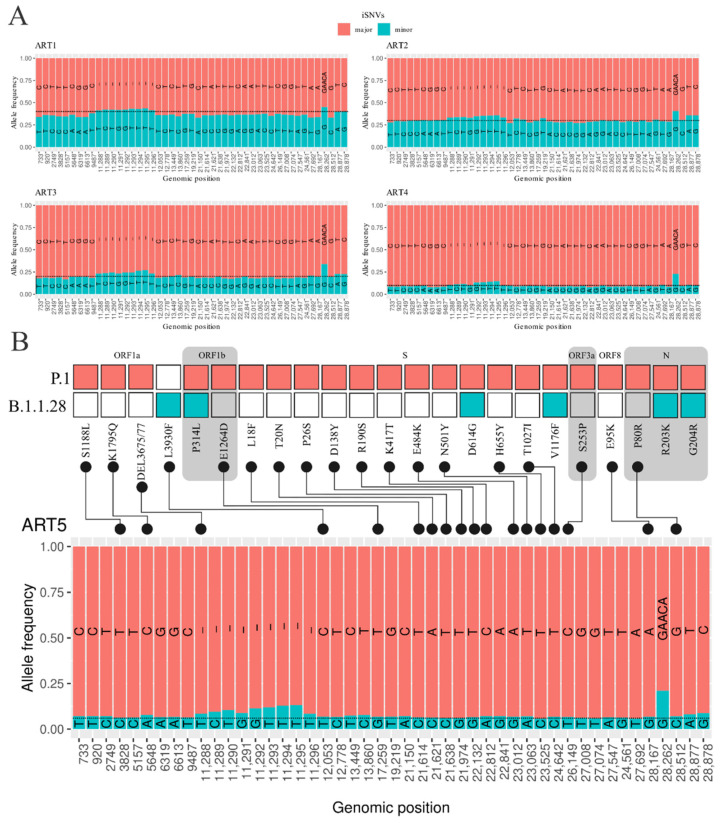
The iSNV frequency sites of artificial datasets simulating co-infection events (ART1 to ART5). The black dashed line represents the expected minor iSNV average frequencies in each artificial dataset. (**A**). The iSNV frequencies of four artificial datasets. (**B**). The lineage-defining mutations of P.1 and B.1.1.28 lineages (upper section) and the allele frequencies of minor and major consensus genomes (lower section). The grey boxes in section (**B**) depict the boundaries of adjacent SARS-CoV-2 proteins bearing lineage-defining mutations.

We detected a low number of iSNVs, from zero to two, with a mean of zero (stdev = 0.43; for details, see Table S2), among the 86 “non-artificial” samples, supporting published estimates of low intrahost variant variability of SARS-CoV-2 [19,32]. To evaluate the performance of the workflow on samples with a large number of iSNVs, we generated five artificial coinfection/codetection samples (see the Section 2). The workflow was able to consistently detect a large amount of well-supported iSNVs (47 iSNVs per sample) (Figure 3A, Table S3). These results show the capacity of the workflow to rapidly detect and generate a range of useful information that is important to generate new insights, such as single consensus and coinfection of different SARS-CoV-2 lineages in a single sample. Moreover, the intrahost multi-allele frequencies can also be used to detect sample contamination in a scenario where most samples show the same intrahost pattern found in a negative control sample.

### 3.3. Detection of Coinfection Events

**Figure 4:**
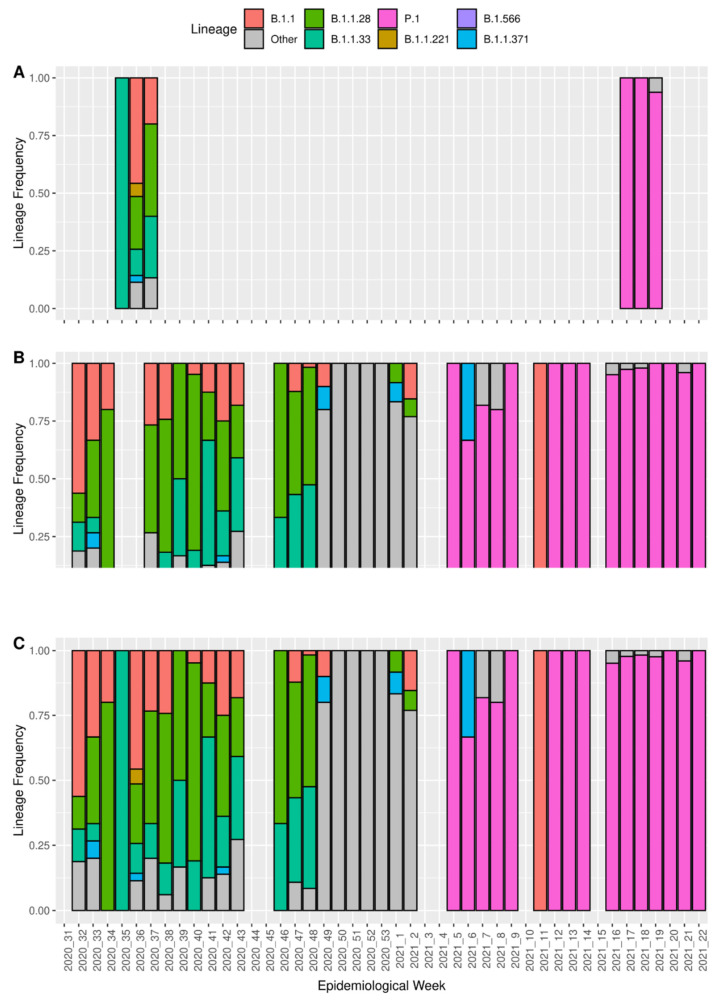
The lineages reported by PANGO version 3.1.11 implemented inside ViralFlow 0.0.6. (**A**). The lineages from the 86 samples used to test viral flow. (**B**). The lineages from 1516 genomes available at the GISAID database (accessed on 30 August 2021) except for the 86 samples used to test ViralFlow. (**C**). The compilation of all genomes available from GISAID (1516) including all 86 samples used to test ViralFlow.

Two key pieces of information are necessary for the deployment of SARS-CoV-2 outbreak control strategies: the virus lineage assignment and the mutation characterization [9,33]. The workflow generates two tabular files for each sample containing this key information: ‘.pango.csv’ and ‘.nextclade.csv’, which provide the virus lineage and the mutations found, respectively. The lineages identified in the 86 non-artificial samples (Figure 4) correspond to the set of mutations and to the expected lineages circulating at the sampling date in each location (Table S4). For the five artificial samples, the virus lineages and set of mutations correspond to the allele frequencies present in iSNV multi-allele frequencies (Figure 3B, Table S3). To show the precision of our workflow in detecting *indel* regions, five random non-artificial samples were assigned as P.1 with the deletion of 11288–11297 in ORF1a and an insertion into the intergenic region at 28,262 positions, which were manually investigated using Integrative Genomics Viewer [34] (Supplementary File S2).

### 3.4. Additional Quality-Check Results

In addition to the intrahost, lineage and mutations information generated by different tools present in our workflow, the ‘.fastp.html’ generated by fastp and the ‘coverage.report’ and ‘chromosomes.report’ files generated by bamdst can be used to assess the quality of mapping and assembly steps and for feedback to wet lab staff. This information can also be crossed with the information of the ‘qc.overallScore’ and ‘qc.overallStatus’ columns present in the ‘.nextclade.csv’ table.

### 3.5. Benchmark

The comparison with HAVoC showed a similar genome coverage (Table S5) between ViralFlow (coverage mean 99.70 with stdev equal to 3.24), HAVoC Test I (coverage mean 99.71 with stdev equal to 2.9) and HAVoC Test II (coverage mean 99.71 with stdev equal to 2.71). The mean depth between ViralFlow and HAVoC Test I are similar (342.2 (stdev equal to 66.47) and 341.54 (stdev equal to 67.42), respectively), and HAVoC Test II showed a high mean depth (404.19 with stdev equal to 80.76). The higher depth of HAVoC Test II can be explained by the lower quality threshold than those used in ViralFlow and HAVoC Test I.

When investigating the consensus genomes, we noticed that HAVoC fails to assemble a deletion region of nine nucleotides in ORF1ab of Gamma (P.1) genomes. In the 34 samples with this deletion, the HAVoC Test I fails to correctly assemble all 34 samples bearing this deletion (Supplementary File S3A), and the HAVoC Test II correctly assembled only 12 of the 34 samples (Supplementary File S3B). Moreover, we found 23 samples with pangolin lineage incongruencies between ViralFlow and HAVoC Test I (Table S5), where HAVoC fails to recover a mutation in position 22,812 of the SARS-CoV-2 genome, probably owing to the presence of minor iSNVs in these genomic loci that support the reference base. In these cases, we performed a manual curation to replace the iSNV with a minor frequency to an iSNV with a major frequency. After the curation, the pangolin signed the correct lineage (Supplementary File S3C and Table S5).

## 4. Conclusions

ViralFlow stands up as a versatile and scalable choice for research groups that work with Illumina paired-end data and need rapid deployment and information processing for SARS-CoV-2 amplicon sequencing data. Our workflow includes reports on the quality of sequencing experiments, quality of consensus genome, and the lineage and mutation profiles that could be easily used in genomic and epidemiological reports. Finally, ViralFlow scales well according to computational resources and is able to deliver results in a few minutes for real-life sequencing batches, even using a modest computational infrastructure.

## Data Availability

The code and workflow documentation is available on https://github.com/dezordi/ViralFlow, accessed on 19 January 2022. The FastQ files of the 86 samples are publicly available on EMBL-EBI study accession PRJEB47823.

## Supplementary Materials

The supplementary data are available at https://www.mdpi.com/article/10.3390/v14020217/s1. Table S1: Artificial samples information; Table S2: Metadata, PANGO lineages and aminoacid mutations per sample; Table S3: Number of MAFs per sample; Table S4: Intrahost information; Table S5: Comparrison between HAVoC (last update 2021-09-03) and ViralFlow 0.0.6; File S1: Artificial dataset created with the ART; File S2: IGV and alignment of artificial samples; File S3: Alignment and IGV of 86 samples processed by HAVoC
