# Supplementary material for: ViralFlow: A Versatile Automated Workflow for SARS-CoV-2 Genome Assembly, Lineage Assignment, Mutations and Intrahost Variant Detection
Source: Viruses. 2022 Jan 23;14(2):217. doi: 10.3390/v14020217 (PMC8877152; doi:10.3390/v14020217)
Supplement: Supplementary file 1 [file viruses-14-00217-s001.zip › supplementary_file_2.pdf]

A

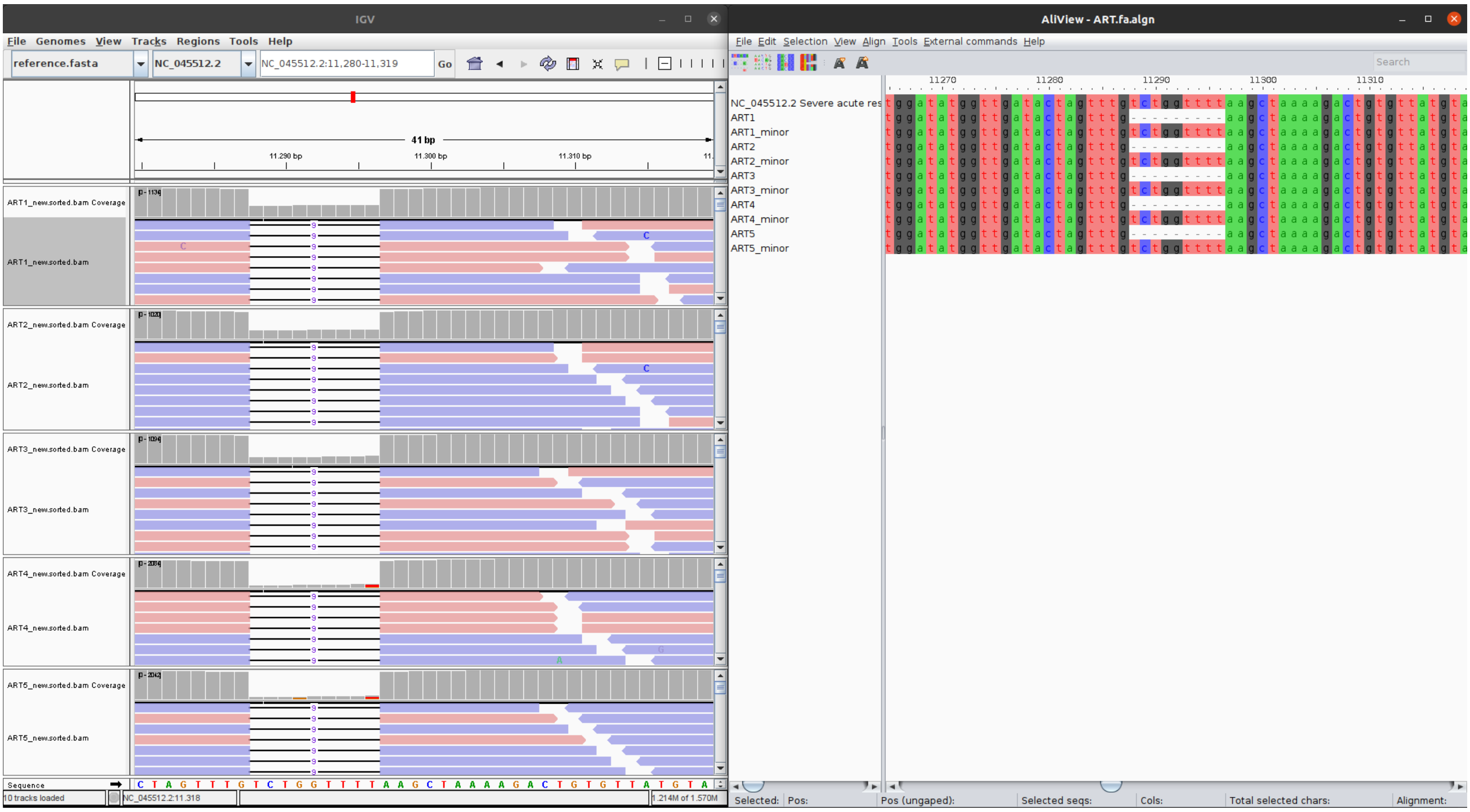

B

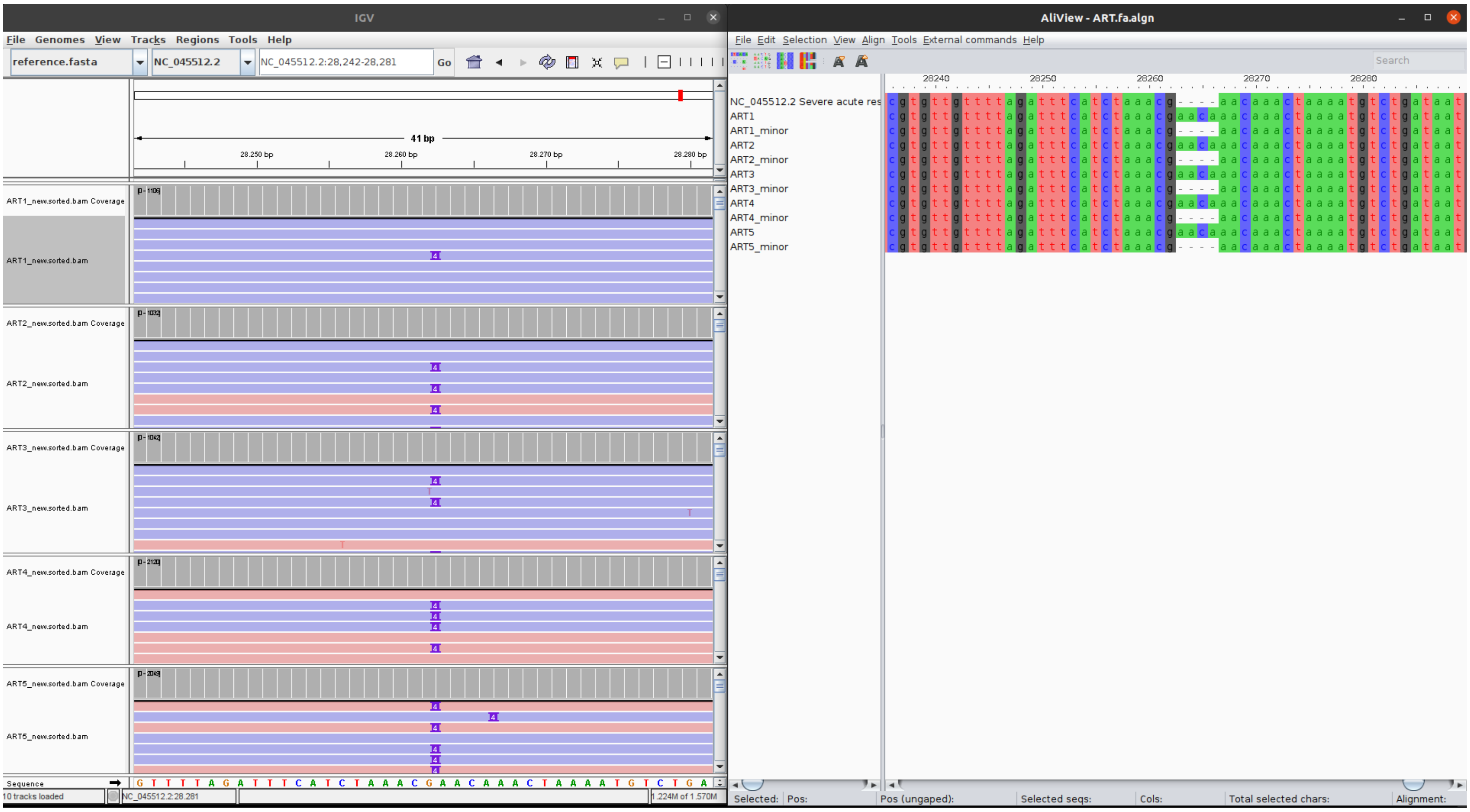

Supplementary File S2 : IGV and alignment of artificial samples. A. Deletion present on ORF1a, genomic position 11288. B. Inserion of the intergenic region, genomic position 28262. The indels present on consensus genomes follow the proport-ion of iSNVs observed, where the indels are present in a major proportion, and the absence of indels are present in minor proportion.
