# Supplementary material for: ViralFlow: A Versatile Automated Workflow for SARS-CoV-2 Genome Assembly, Lineage Assignment, Mutations and Intrahost Variant Detection
Source: Viruses. 2022 Jan 23;14(2):217. doi: 10.3390/v14020217 (PMC8877152; doi:10.3390/v14020217)
Supplement: Supplementary file 1 [file viruses-14-00217-s001.zip › supplementary_file_3.pdf]

A

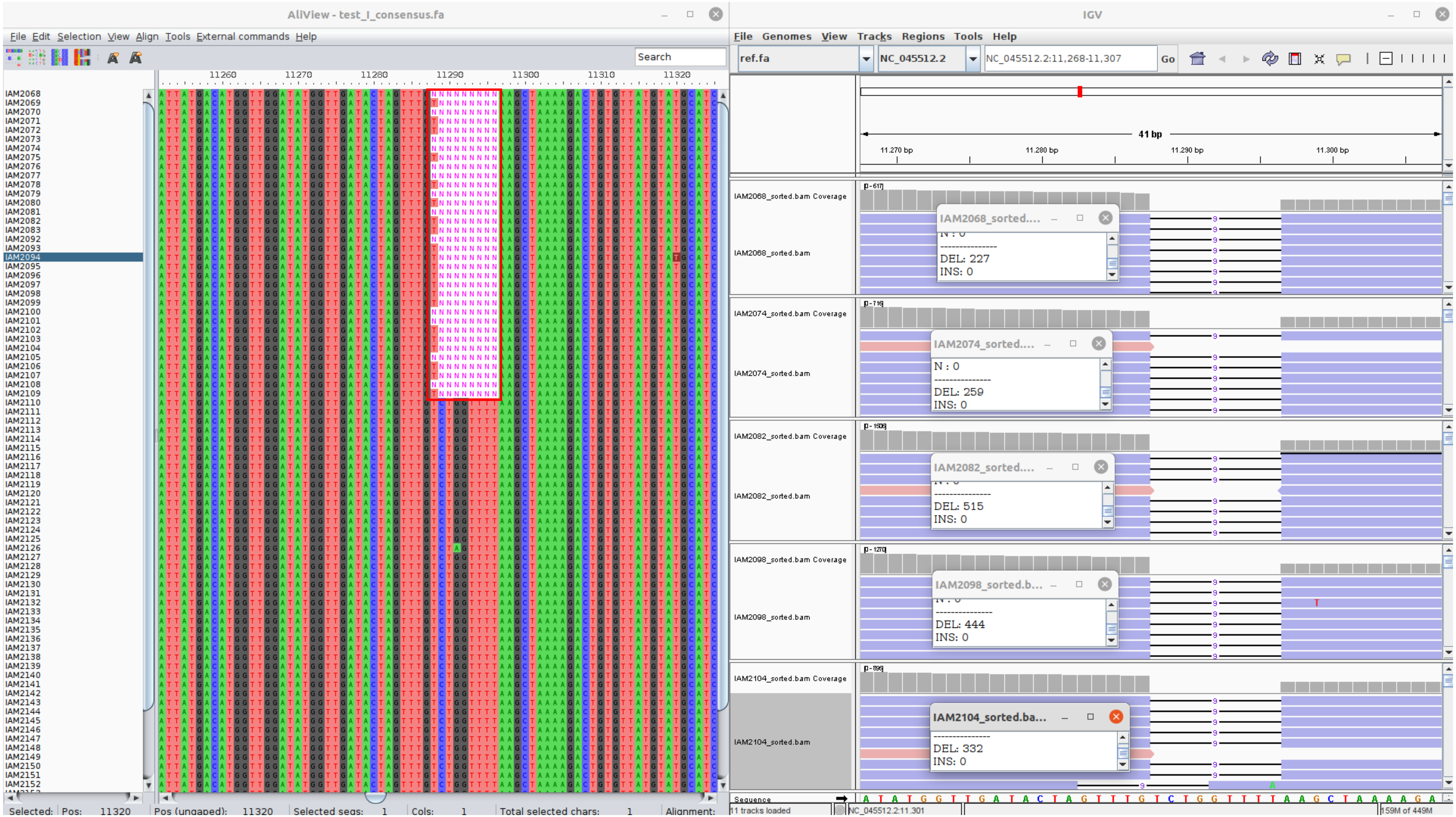
AliView - test_I_consensus.fa
File Edit Selection View Align Tools External commands Help
Search
IGV
File Genomes View Tracks Regions Tools Help
ref.fa
NC_045512.2
NC_045512.2:11,268-11,307
Go
41 bp
IAM2068_sorted.bam Coverage
IAM2068_sorted.bam
DEL: 227
INS: 0
IAM2074_sorted.bam Coverage
IAM2074_sorted.bam
N : 0
DEL: 259
INS: 0
IAM2082_sorted.bam Coverage
IAM2082_sorted.bam
DEL: 515
INS: 0
IAM2098_sorted.bam Coverage
IAM2098_sorted.bam
DEL: 444
INS: 0
IAM2104_sorted.bam Coverage
IAM2104_sorted.bam
DEL: 332
INS: 0
Sequence
11 tracks loaded
NC_045512.2:11.301
159M of 449M
Selected: Pos: 11320
Pos (ungaped): 11320
Selected seqs: 1
Cols: 1
Total selected chars: 1

B

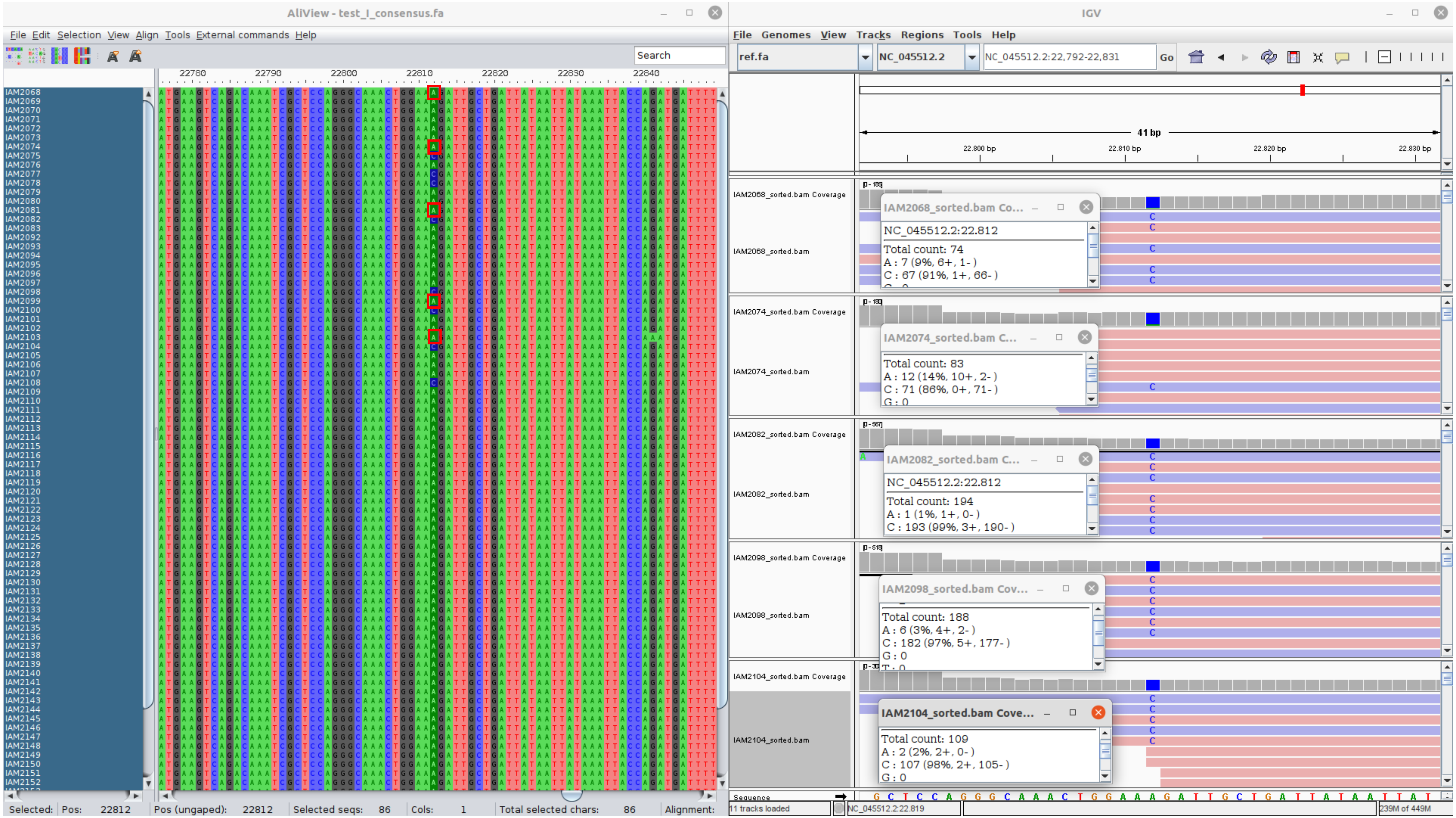
AliView - test_I_consensus.fa
File Edit Selection View Align Tools External commands Help
Search
22780 22790 22800 22810 22820 22830 22840
Selected: Pos: 22812 Pos (ungaped): 22812 Selected seqs: 86 Cols: 1 Total selected chars: 86 Alignment:
IGV
File Genomes View Tracks Regions Tools Help
ref.fa
NC_045512.2
NC_045512.2:22,792-22,831
Go
41 bp
22.800 bp
22.810 bp
22.820 bp
22.830 bp
IAM2068_sorted.bam Coverage
IAM2068_sorted.bam
IAM2068_sorted.bam Co...
NC_045512.2:22.812
Total count: 74
A : 7 (9%, 6+, 1-)
C : 67 (91%, 1+, 66-)
IAM2074_sorted.bam Coverage
IAM2074_sorted.bam
IAM2074_sorted.bam C...
Total count: 83
A : 12 (14%, 10+, 2-)
C : 71 (86%, 0+, 71-)
IAM2082_sorted.bam Coverage
IAM2082_sorted.bam
IAM2082_sorted.bam C...
NC_045512.2:22.812
Total count: 194
A : 1 (1%, 1+, 0-)
C : 193 (99%, 3+, 190-)
IAM2098_sorted.bam Coverage
IAM2098_sorted.bam
IAM2098_sorted.bam Cov...
Total count: 188
A : 6 (3%, 4+, 2-)
C : 182 (97%, 5+, 177-)
G : 0
IAM2104_sorted.bam Coverage
IAM2104_sorted.bam
IAM2104_sorted.bam Cove...
Total count: 109
A : 2 (2%, 2+, 0-)
C : 107 (98%, 2+, 105-)
G : 0
Sequence
11 tracks loaded
NC_045512.2:22.819
239M of 449M

C

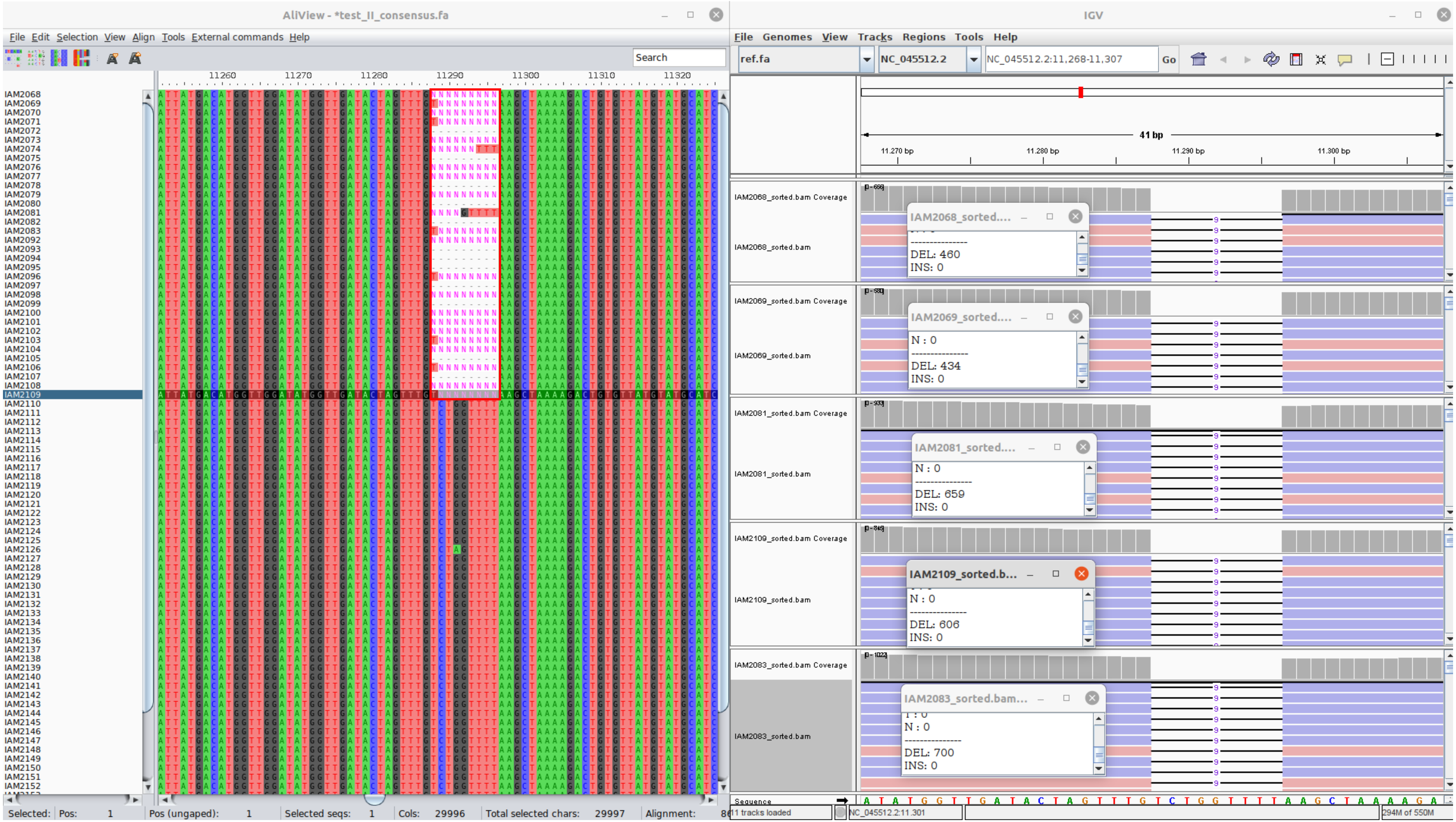

Supplementary File S3 : Alignment and IGV of 86 samples processed by HAVoC. **A**. The ORF1ab 11288 region of HAVoC Test I; **B**. The 22812 region of HAVoC Test 1; **C**. The ORF1ab 11288 region of HAVoC Test II. In red the investigated regions, for each example, 5 random bam files were selected.
